# BRCA1 prevents R-loop-associated centromeric instability

**DOI:** 10.1038/s41419-021-04189-3

**Published:** 2021-10-01

**Authors:** Carine Racca, Sébastien Britton, Sabrine Hédouin, Claire Francastel, Patrick Calsou, Florence Larminat

**Affiliations:** 1 Institut de Pharmacologie et Biologie Structurale, IPBS, Université de Toulouse, CNRS, UPS, Toulouse, France; 2 Equipe Labellisée Ligue contre le Cancer, 2018 Toulouse, France; 3 Université de Paris, Epigénétique et Destin Cellulaire, CNRS, Paris, F-75013 France; 4 Present Address: Basic Sciences Division, Fred Hutchinson Cancer Research Center, Seattle, WA USA

**Keywords:** Centromeres, Double-strand DNA breaks

## Abstract

Centromeres are defined by chromatin containing the histone H3 variant CENP-A assembled onto repetitive α-satellite sequences, which are actively transcribed throughout the cell cycle. Centromeres play an essential role in chromosome inheritance and genome stability through coordinating kinetochores assembly during mitosis. Structural and functional alterations of the centromeres cause aneuploidy and chromosome aberrations which can induce cell death. In human cells, the tumor suppressor BRCA1 associates with centromeric chromatin in the absence of exogenous damage. While we previously reported that BRCA1 contributes to proper centromere homeostasis, the mechanism underlying its centromeric function and recruitment was not fully understood. Here, we show that BRCA1 association with centromeric chromatin depends on the presence of R-loops, which are non-canonical three-stranded structures harboring a DNA:RNA hybrid and are frequently formed during transcription. Subsequently, BRCA1 counteracts the accumulation of R-loops at centromeric α-satellite repeats. Strikingly, BRCA1-deficient cells show impaired localization of CENP-A, higher transcription of centromeric RNA, increased breakage at centromeres and formation of acentric micronuclei, all these features being R-loop-dependent. Finally, BRCA1 depletion reveals a Rad52-dependent hyper-recombination process between centromeric satellite repeats, associated with centromere instability and missegregation. Altogether, our findings provide molecular insights into the key function of BRCA1 in maintaining centromere stability and identity.

## Introduction

Centromeres are the essential chromatin domains which coordinate the assembly of kinetochores, the proteinaceous complexes required for the attachment of the spindle microtubules to chromosomes during mitosis. Dysfunction of the centromere/kinetochore machinery results in numerical (e.g., trisomy) and structural chromosome instability (e.g., translocations), which influences fertility and oncogenesis. Hence, maintaining centromere integrity is key to faithfully transmit genetic information during cell division and prevent chromosome segregation errors which have important implications in human health.

Human centromeres are genetically defined by the presence of large arrays of tandem repeats known as α-satellite (α-SAT) sequences, which extend over several mega-bases with a chromosome-specific composition and show greater sequence divergence among eukaryotes than telomeres [1, 2]. These repetitive sequences are generally unstable and prone to DNA breaks and rearrangements which can cause chromosome instability [3]. Centromeres are also epigenetically defined through the deposition of the histone H3 variant CENP-A into a subset of nucleosomes [4]. Far from being transcriptionally inert, centromeric repeats are actively transcribed in most species by RNA polymerase II (RNAPII) [5]. The resulting non-coding centromeric transcripts (cenRNAs) are integral parts of CENP-A-containing chromatin and participate in kinetochore assembly at the onset of mitosis [6–8]. Centromeric transcripts are present at the centromere of every chromosome and are abundant throughout the cell cycle [9]. However, unscheduled accumulation of cenRNAs is associated with centromeric instability and leads to defects in chromosome segregation [10]. In addition, RNAPII-dependent transcription could represent a threat to centromere integrity through the formation of co-transcriptional R-loops. R-loops are transient structures which form frequently during transcription, when the nascent transcript anneals to the complementary DNA template strand. This produces three-stranded nucleic acid structures composed of a DNA:RNA hybrid and a displaced single-stranded DNA [11, 12]. R-loops can be resolved by members of the RNase H family, which specifically degrade the RNA in DNA:RNA hybrids [13], or by DNA–RNA helicases, like senataxin (SETX) which unwinds the hybrid and allows access of the 5′-3′ exonuclease Xrn2 to promote transcription termination [14]. R-loops are involved in a variety of physiological processes, including transcription [15], DNA repair [16], and chromosome segregation [17], but are also a source of DNA damage and genome instability upon the loss of an R-loop suppressor [18]. Many studies have demonstrated that co-transcriptional RNA:DNA hybrids are a major obstacle to replication fork progression, contributing to replication stress [19]. Interestingly, R-loops favor repeats instability in disease-causing repetitive sequences like in Huntington’s disease and Fragile X syndrome [20]. This suggests that R-loops formation and processing may also promote centromeric repeats instability and raises the question of the mechanisms which protect centromeres from deleterious R-loops accumulation.

BRCA1 is a tumor suppressor involved in many of the processes required to ensure chromosomal stability [21], including the activation of cell cycle checkpoints, repair of DNA double-strand breaks (DSBs) by homologous recombination (HR), stabilization of stalled replication forks, and processing of R-loops at sites of transcriptional pausing [22, 23]. In undamaged cells, BRCA1 is distributed in discrete foci [24], a subset of which is associated with centromeres during both interphase [25] and mitosis [26]. Likewise, constitutive genomic occupancy of BRCA1 at centromere regions was detected throughout the cell cycle [26, 27]. Moreover, a high-resolution interaction neighborhood map of BRCA1 identified several centromere-associated proteins in its close proximity [28] and BRCA1 loss leads to an accumulation of cenRNAs [27]. We previously reported that BRCA1 deficiency weakens centromere cohesion during prometaphase, reduces accumulation of Aurora B kinase at the inner centromere and promotes a defect in chromosome segregation [26]. Despite these links between BRCA1 and centromere, it is still unknown what drives the localization of BRCA1 at centromeres and how BRCA1 contributes to centromere stability in undamaged cells. Here we show that BRCA1 maintains the centromere identity and integrity by preventing local R-loop accumulation and associated DNA damage.

## Materials and methods

### Cell culture

Human fibrosarcoma HT1080 and osteosarcoma U-2OS cell lines as well as the HB-8730 hybridoma cell line which produces the monoclonal S9.6 IgG were obtained from the American Type Culture Collection (ATCC) and maintained in Dulbecco’s Modified Eagle’s Medium (DMEM) with GlutaMax (Gibco) supplemented with 10% fetal calf serum (FCS, Lonza), 1 mM sodium pyruvate (Gibco), and 1% penicillin-streptomycin (Gibco). BRCA1 mutated-breast cancer HCC1937 cell line [29] and wtBRCA1-complemented HCC1937 stable cell line were kindly provided by J. Chen (University of Texas, MD Anderson Cancer Center, Houston, USA). Both were grown in RPMI-1640 medium with GlutaMAX supplemented with 10% FCS, 1 mM sodium pyruvate, 10 mM HEPES, and 1% penicillin-streptomycin. wtBRCA1 expression was maintained in HCC1937 cells using selective medium containing 200 μg/ml G418. U-2OS T-Rex cells expressing a doxycycline-inducible RNAse HI-NLS-mCherry were described previously [30]. They were grown in DMEM medium supplemented with 10% FCS, 1% penicillin-streptomycin and 0.2 μg/ml puromycin. Expression of RNAse HI-mCherry was achieved using 2 μg/ml doxycycline (Merck) for 8 h within the DMEM medium. ΔRad52 U-2OS cells [31] were kindly provided by T. Yasuhara (University of Tokyo, Center for Disease Biology and Integrative Medicine, Tokyo, Japan). All cells were grown in a humidified atmosphere at 37 °C with 5% CO_2_ and checked for absence of mycoplasma contamination.

### S9.6 purification and cell transfection and treatment

The purification of the S9.6 IgG from HB-8730 culture supernatants was performed using HiTrap Protein G columns (GE Healthcare). The antibody was eluted with 100 mM glycine (Sigma-Aldrich) pH 2.5 in 500 μl fractions. Fractions were assessed for antibody presence by SDS-PAGE and were first dialyzed against PBS overnight and then against 30% glycerol in PBS for 4 h. The antibody concentration was measured using a NanoDrop spectrophotometer (Thermo Fisher Scientific) at 280 nm.

siRNAs transfections were carried out using INTERFERin (Polyplus Transfection) following the manufacturer’s instructions and were performed twice within 24 h of each other using 20 nM siRNA (final concentration) diluted in OptiMEM (Invitrogen). All siRNAs used in the study were purchased from Eurofins Genomics (Ebersberg). The following siRNAs were used: control siRNA, UAGCGACUAAACACAUCAA; siBRCA1 pool: CAACAUGCCCACAGAUCAA, CCAAAGCGAGCAAGAGAAU, UGAUAAAGCUCCAGCAGGA, GAAGGAGCUUUCAUCAUUC; siCENP-A: GGACUCUCCAGAGCCAUGA; siSETX: GAAGAGUACUUUGGUCGAUAA. All following experiments were performed 24 h after the second siRNA transfection.

Plasmid transfections were carried out using Lipofectamine 2000 (Thermo Fisher Scientific) following the manufacturer’s instructions. GFP-RNase H1 is a kind gift from R. Crouch (NIH, Bethesda, MD, USA) and GFP-nuc was purchased from Invitrogen. For cell sorting, cells were resuspended in PBS containing 10% FBS and sorted on a BD FACSAria Fusion cell sorter (BD Biosciences). Immunostaining experiments were performed 24 h after plasmid transfection. To inhibit RNA Pol II, cells were incubated overnight with 2 μM α-amanitin (Merck) prior to performing the immunostaining.

Ionizing radiation treatment corresponding to a 1 Gy X-Ray irradiation was performed using a calibrated X-ray irradiator (Faxitron RX-650) followed by a 30 min post-incubation at 37 °C.

### DRIP assay

DNA:RNA immunoprecipitation assay was adapted from previous studies [22, 32, 33]. Briefly, genomic DNA was extracted from 5 million cells using a genomic DNA purification kit (Macherey-Nagel) according to the manufacturer’s protocol. DNA was resuspended in 200 μl of elution buffer (5 mM Tris-HCl pH 8) and then fragmented on ice by sonication using a microtip for 2 × 10 s (Branson Sonifier 250; power setting of 5, 50% duty cycle; 10 sec ON, 10 sec OFF) to yield an average fragment size of 800–300 bp. Chromatin fragment size was monitored by agarose gel electrophoresis. Half of each sample was treated with *E. coli* RNase HI (New England Biolabs) for 2 h at 37 °C in 1x RNase H reaction buffer. The other half was mock-incubated. After setting aside 1% for input DNA, 2 μg of DNA was used for immunoprecipitation with 400 μl binding buffer (10 mM NaPO_4_, pH 7.0/140 mM NaCl/0.05% Triton X-100) and 5 μg of the S9.6 antibody (Kerafast, 1 mg/ml or produced in house) on a rotative shaker at 4 °C for at least 4 h. At the end of the incubation, 25 μl of protein A magnetic beads (Diagenode) prewashed 2−3 times with binding buffer were added to the DNA/antibody complex and incubated for at least 4 h at 4 °C on a rotative shaker. After four washes with 1 ml binding buffer at 4 °C for 10 min each, the beads were eluted using 100 μl of DNA isolation buffer (Diagenode) containing 1 μl proteinase K (20 mg/ml). They were incubated for 15 min in a thermomixer (Eppendorf) at 55 °C at a mixing speed of 800 rpm, and then for 15 min at 100 °C at 800 rpm. The eluted DNA was analyzed by qPCR using the comparative C_T_ method. The DNA:RNA hybrid enrichment was calculated based on the IP/input ratio. All graphs in this study were generated with GraphPad Prism.

### Chromatin immunoprecipitation (ChIP) assay

ChIP assays were performed using 10 million cells/IP. Cells were cross-linked in medium containing 1% formaldehyde at room temperature for 10 min with rotation. Formaldehyde was quenched by the addition of glycine (final concentration of 125 mM) for 5 min. Cell lysis, nuclei isolation, and immunoprecipitation (IP) were performed using the HighCell ChIP kit following the manufacturer’s recommendations (Diagenode). Chromatin fractions were sheared on ice for 10 × 10 s using a microtip (Branson Sonifier 250; power setting of 5, 50% duty cycle, 10 sec ON, 10 sec OFF) to yield a DNA fragment size <1000 bp. Chromatin fragment size was monitored by agarose gel electrophoresis after DNA purification. After dilution of chromatin in ChIP buffer complemented with protease and phosphatase inhibitors cocktail (Thermo Fischer Scientific), samples were incubated overnight at 4 °C with the indicated relevant or control antibodies bound to 25 μl Protein A-coated magnetic beads (Diagenode) (Table S1 for details on antibodies). Beads were captured using a magnetic rack and sequentially washed before elution and DNA purification. Relative quantitation of target sequences in the input and the IP chromatin was performed by qPCR. The fold enrichment of a protein associated to a specific sequence was calculated with respect to the input DNA (1% of the ChIP fraction) and was compared with a ChIP signal obtained using a control non-relevant IgG.

### Real-time PCR (qPCR)

qPCR was performed using the SsoFast EvaGreen Supermix (Bio-Rad) supplemented with 0.4 μM specific primer pairs (sequences of primers are listed in Table S2) and a CFX96 cycler (Bio-Rad). Each qPCR reaction was performed in technical duplicate. All experiments included a standard curve for each primer pair used. For the copy number variation assay, copy number of the target sequence was determined using the comparative Ct(ΔΔCt) calculation method and the Top3 gene as reference sequence. All results were analyzed using Bio-Rad Quantity One analysis software.

### RNA extraction and real-time reverse transcription PCR

Total RNA was isolated using the RNeasy Plus kit (Qiagen) according to the manufacturer’s instructions. RNA was subsequently treated with a TURBO DNase (ThermoFisher Scientific). RT-qPCR was performed in one step using 10 ng of total RNA and the iTaq Universal SYBR Green One-Step kit (Biorad) supplemented with 0.3 μM specific primer pairs (sequences of primers are listed in Table S2) and a CFX96 cycler (Bio-Rad). Each RT-qPCR reaction was performed in technical triplicate. All experiments included a control reaction without reverse transcriptase. Copy number of the target sequence was determined using the comparative Ct(ΔΔCt) calculation method and the GAPDH gene as reference sequence. All results were analyzed using Bio-Rad Quantity One analysis software.

### Immunofluorescent staining, imaging, and analysis

Cells were pre-extracted, fixed, and permeabilized as previously described [26], except for the immunostaining of S9.6 in combination with CREST in which samples were prepared using a protocol recently reported [34]. Incubation with relevant primary and secondary antibodies was carried out sequentially for 1 h each at room temperature (Table S1). Single plane and z-stack images were captured using a confocal laser microscope (FV1000 Olympus) with a Plan-Apochromat 60x NA 1.40 oil immersion lens or a Plan-Apochromat 40x NA 0.95 lens and 405-, 473-, 559- and 635-nm laser excitation. The sequential mode was used to acquire images without cross-talk. When comparing experimental conditions, images were taken using the same exposure conditions. Image processing such as maximal-intensity projections of the z-series and quantification were performed using ImageJ software (NIH). For CENP-A or γH2AX signals, a mask was generated to mark all centromeres defined by CREST immunostaining in the projected image. After background subtraction, the mean intensities of signals in the mask were measured.

### Chromosome orientation fluorescence in situ hybridization (CO-FISH) methodology

The CO-FISH assay was adapted from previous studies [35, 36]. Briefly, exponentially growing cells were cultured overnight in the presence of 10 μM BrdU:BrdC (3:1) (Sigma-Aldrich) at 37 °C to allow for one round of replication. Colcemid (Roche) was then added at a concentration of 0.1 μg/ml for 4 h to arrest cells at prometaphase. After fixation of cellular preparations on slides and Hoechst 33258 (Sigma-Aldrich) staining, the newly synthesized strands were degraded following UV light exposure and treatment with 10 U/μl ExoIII (Promega). Hybridization was performed using fluorescent centromeric PNA probes against CENP-B box motif sequences. The PNA probe labeled with Cy3 (ATTCGTTGGAAACGGGA; PNABio Inc) hybridizes with the leading strand and the reverse PNA probe labeled with Alexa-488 (TCCCGTTTCCAACGAAT; Eurogentec) hybridizes with the lagging strand. DNA was counterstained with DAPI (Sigma-Aldrich). Metaphases were captured on a confocal laser microscope (FV1000 Olympus) and images were analyzed using Image J software (NIH). Quantitation to measure for SCE between α satellite sequences (C-SCE) was done by counting the number of CO-FISH signals showing C-SCE over the total number of CO-FISH signals observed for each metaphase.

### Click-iT chemistry

Nascent RNA transcripts were labeled by EU incorporation (1 mM for 1 h) and detected using click reaction with Alexa Fluor 488 Imaging kit (Thermo Fischer Scientific) according to the manufacturer’s instructions.

### Statistical analysis

Results are presented with significance calculated by Mann–Whitney U test or Student’s *t* test with standard software (GraphPad Prism, GraphPad Software). Significance was assigned for a *p*-value < 0.05.

## Results

### BRCA1 occupancy at centromeres relies on R-loops

**Fig. 1:**
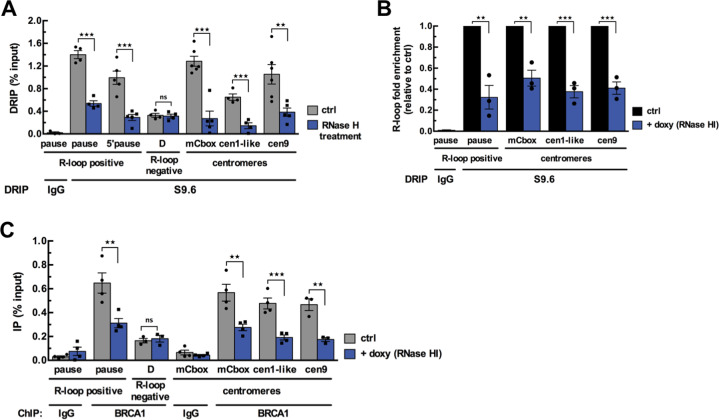
BRCA1 enrichment at centromeres is promoted by R-loops. **A** DRIP-qPCR analysis performed in U-2OS cells at R-loop-positive and -negative loci of the β-actin gene and at α-SAT repeats of different centromeres. RNase H treatment was carried out on half of each sample before the IP. The graph shows the R-loop enrichment (as percent input) as mean ± SEM. *n* = 4 to 6 independent experiments. **p < 0.01, ***p < 0.001, ns: not significant (Mann–Whitney test). **B** DRIP-qPCR analysis performed in U-2OS T-Rex cells expressing a doxycycline-inducible RNAse HI-NLS-mCherry. Graph shows the R-loop fold enrichment normalized to control conditions (ctrl) following RNAse HI overexpression (+ doxy) as mean ± SEM. *n* = 3 independent experiments. **p < 0.01, ***p < 0.001 (Mann–Whitney test). **C** BRCA1 ChIP experiment performed in U-2OS T-Rex cells expressing a doxycycline-inducible RNAse HI-NLS-mCherry. Graph shows BRCA1 fold enrichment (as percent input) in control conditions (ctrl) and following RNAse HI overexpression (+ doxy) as mean ± SEM. *n* = 3–4 independent experiments. **p < 0.01, ***p < 0.001, ns: not significant (Mann–Whitney test).

We first assessed whether α-SAT repeats are prone to R-loop formation at human centromeres using asynchronous U-2OS and HT1080 cancer cells. We performed a well-established DNA:RNA hybrid immunoprecipitation (DRIP) assay using the S9.6 antibody directed against the hybrids [37], followed by qPCR to amplify specific centromeric α-SAT arrays (mCbox, cen1-like and cen9) from different chromosomes [38–40]. Genomic DNA treated with RNase H prior to DRIP served as a control to ensure signal specificity. We used positive and negative test regions as readouts of RNA:DNA hybrid formation including two RNAPII pause sites downstream of the coding region of the β actin gene (R-loop positive pause and 5′ pause site probes), where high levels of R-loops have been reported, and a negative region (D probe) with no detectable R-loops [14] (Fig. S1A). R-loops were detected in all the centromeric α-SAT arrays tested in both cell lines (Fig. 1A and Fig. S1B) and the DRIP signals were significantly reduced by pretreatment with RNase H (Fig. 1A and Fig. S1B). Analysis of the in situ pattern of S9.6 staining in interphase nuclei of U-2OS cells confirmed that several centromeres (CREST staining) displayed R-loop foci (Fig. S1C). α−amanitin treatment, which strongly decreases nascent transcripts synthesis (Fig. S1D) [41], reduced centromeric R-loop signal (Fig. S1C). In addition, since the levels of R-loops can be manipulated in cellulo using RNAse H overexpression [42], we engineered U-2OS cells to inducibly express ectopic RNase HI protein fused to the fluorescent mCherry [30] (Fig. S1E). Overexpression of RNase HI led to reduced R-loop signals measured at centromeric α-SAT by DRIP-qPCR (Fig. 1B). Altogether these data show that transcription-dependent R-loops form naturally in α-SAT arrays of interphase human cells.

We next investigated whether R-loop formation triggered BRCA1 localization at centromeres. Chromatin immunoprecipitation (ChIP) analysis revealed first, that BRCA1 was constitutively present on all α-SAT arrays tested and second, that BRCA1 occupancy at centromeric chromatin was reduced more than 2-fold under conditions where R-loop abundance was significantly decreased by RNase HI overexpression (Fig. 1C). RNase H overexpression also reduced the binding of SETX, an established BRCA1 partner and R-loop processing factor (Fig. S1F). These data strongly support the view that, in undamaged cells, BRCA1 localization at centromeres is promoted by the presence of R-loops.

### BRCA1 counteracts R-loop accumulation at centromeres

**Fig. 2:**
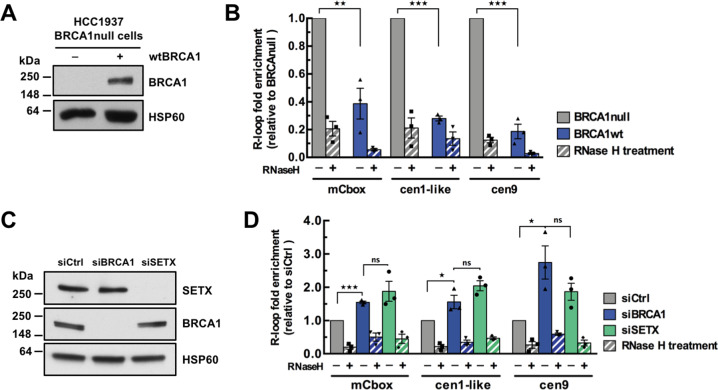
BRCA1 counteracts R-loop accumulation at centromeres. **A** Immunoblot of whole-cell lysates reflecting BRCA1 rescue in wtBRCA1-reconstituted HCC1937 cells. HSP60 was used as a loading control. **B** DRIP-qPCR analysis performed in HCC1937-derived cell lines. Graph shows the R-loop fold enrichment normalized to BRCA1-null conditions as mean ± SEM. *n* = 3 independent experiments. RNase H treatment was carried out before the IP to demonstrate specificity of the signal. **p < 0.01, ***p < 0.001 (Mann–Whitney test). **C** Immunoblot of whole-cell lysates showing the efficiency of the siRNA-mediated depletion of BRCA1 and SETX in HT1080 cells. HSP60 was used as a loading control. **D** DRIP-qPCR analysis performed in siRNA-treated HT1080 cells. Graph shows the R-loop fold enrichment normalized to control conditions (siCtrl) as mean ± SEM. *n* = 3 independent experiments. RNase H treatment was carried out before the IP to demonstrate specificity of the signal. *p < 0.05, ***p < 0.001, ns: not significant (Mann–Whitney test).

Since the presence of R-loops per se is critical to drive BRCA1 at centromeres, we next addressed whether in turn BRCA1 regulates R-loop levels at centromeres. Here, we compare the breast cancer cell line HCC1937, carrying a protein-truncation mutation in one allele of BRCA1 while the other allele is lost, complemented with an empty plasmid (BRCA1null), with its isogenic counterpart expressing a wild-type BRCA1 cDNA (BRCA1wt) [43] (Fig. 2A). DRIP signals assessed at a known positive R-loop region were obtained with the expected results in these cells (Fig. S2A). Consistent with BRCA1 promoting SETX recruitment to R-loops [22], we found that SETX occupancy at centromeres significantly increased upon wtBRCA1 expression in HCC1937 cells (Fig. S2B). Importantly, the levels of RNase HI-sensitive DNA:RNA hybrids measured by DRIP-qPCR over several centromeres were 2.5- to 5-fold lower in wtBRCA1 cells than in BRCA1null cells (Fig. 2B).

To unambiguously demonstrate the critical role of BRCA1 in the processing of R-loops at centromeres, we also investigated R-loop formation in HT1080 cells depleted of endogenous BRCA1 using RNA interference (Fig. 2C). Depleting BRCA1 did not affect cell cycle progression (Fig. S2C). As expected, the detected DRIP signals were reduced by RNase HI treatment at all centromeric repeats tested (Fig. 2D) and at a common positive control region (Fig. S2E). Under conditions where the level of BRCA1 was efficiently reduced, including at centromeres (Fig. S2D), we observed a 1.4- to 2.8-fold increase in R-loop formation over various α-SAT repeats in BRCA1-depleted cells compared to mock-transfected control cells (Fig. 2D). We further confirmed BRCA1 function in limiting unscheduled centromeric R-loops accumulation by IF analysis of siRNA-transfected U-2OS cells (Fig. S2F). In these cells, BRCA1 depletion led to a 2-fold increase in the number of R-loop foci localized at centromeres (from 6.75 ± 0.85 in ctrl cells to 13.5 ± 1.66 centromeric R-loop foci in BRCA1-depleted cells), while R-loop signal was strongly reduced by α-amanitin treatment (Fig. S2F). Finally, we found that RNAi-mediated depletion of SETX increased R-loops levels at centromeres to the same extent as BRCA1 depletion (Fig. 2D). Therefore, we conclude that BRCA1 counteracts the accumulation of R-loops over centromeric repeats, most likely through the co-recruitment of its partner SETX.

### BRCA1 maintains centromeric chromatin identity

**Fig. 3:**
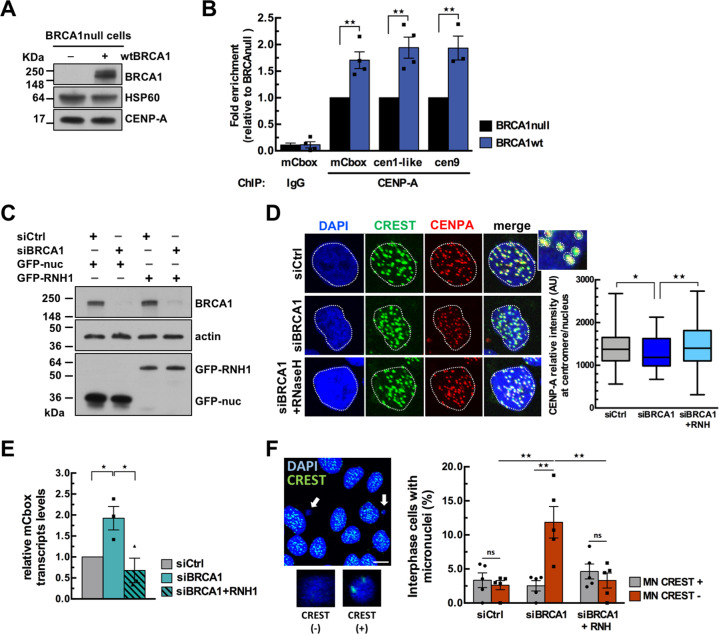
BRCA1 maintains centromeric chromatin identity. **A** Immunoblot of whole-cell lysates showing that BRCA1 rescue in HCC1937 cells does not modulate overall CENP-A levels. HSP60 was used as loading control. **B** CENP-A ChIP experiment performed in HCC1937-derived cell lines. Graph shows CENP-A fold enrichment normalized to BRCA1-null conditions as mean ± SEM. *n* = 4 independent experiments. **p < 0.01 (Mann–Whitney test). **C** Immunoblot of whole-cell lysates showing the efficiency of the siRNA-mediated depletion of BRCA1 and of the expression of GFP-hRNaseH1 in U-2OS cells. Actin was used as loading control. **D** Representative images showing DAPI (blue), anti-centromere Ab CREST (green) and anti-CENP-A Ab (red) signals from transfected U-2OS cells. CENP-A signals were measured at centromeres from maximum intensity projections of Z-series. A mask was generated to mark all centromeres on the basis of CREST staining in the image. The mean intensity of CENP-A in the mask was measured and normalized in each nucleus. The box plot shows the distribution of the mean CENP-A intensity measured at each centromere/nucleus from *n* ≥ 50 cells analyzed for each condition in one biological experiment. **p < 0.01, *p < 0.05 (Student’s *t* test). Similar results were obtained from two additional independent experiments. **E** RT-qPCR analysis of cenRNA transcripts (mCbox) levels in U-2OS cells cells co-transfected with siRNAs and GFP-hRNase H1. Data were normalized to GAPDH and compared to siCtrl and are shown as mean ± SEM. *n* = 3 independent experiments. *p < 0.05 (Mann–Whitney test). **F** Representative immunofluorescence image displaying U-2OS cells stained with DAPI and CREST (green). Two micronuclei (MN) are magnified to illustrate DAPI ( + ) CREST ( + ) and DAPI ( + ) CREST (-) MN. Scale bar, 10 μm. The quantitative analysis shows the mean percentage ± SEM of cells with MN from *n* ≥ 150 cells analyzed for each condition in one biological experiment. *n* = 5 independent experiments. **p < 0.01, ns, not significant (Student’s *t* test).

Evidence suggests that R-loops influence chromatin modifications by affecting nucleosome density and/or via recognition by chromatin regulators [44]. The BRCA1-depleted cells which accumulate DNA:RNA hybrids at α-SAT repeats provide a unique opportunity to assess the consequences of R-loop accumulation on the surrounding chromatin. To address this, we investigated CENP-A occupancy at centromeres in BRCA1null and wtBRCA1-rescued HCC1937 cells. While overall expression levels of CENP-A were similar in both cell lines (Fig. 3A), ChIP analysis indicated that CENP-A occupancy at α-SAT repeats was significantly higher (1.5 to 2-fold) in wtBRCA1 than in BRCA1null cells (Fig. 3B).

We next examined whether this phenotype can be suppressed by RNase H overexpression. We quantify CENP-A staining intensity at centromeres (defined by CREST staining) by IF in U-2OS cells which were co-transfected with siRNA and either a plasmid expressing GFP-hRNase H1 [45] or a nuclear GFP (GFP-nuc) control plasmid. Cells were sorted by flow cytometry to analyze comparable populations of GFP-positive cells (Fig. 3C). The specificity of the CENP-A signal was validated following CENP-A depletion which led to a significant reduction of CENP-A staining in cells (Fig. S3A; S3B). While the overall cellular levels of CENP-A were comparable between control and BRCA1-depleted cells (Fig. S3A), BRCA1 depletion significantly decreased centromeric CENP-A signal by 15–20% (Fig. 3D), Importantly, overexpression of hRNase H1 counteracted the effects of BRCA1 depletion on the reduction of centromeric CENP-A levels (Fig. 3D), supporting the notion that centromeric chromatin identity is tightly linked to R-loop processing.

CenRNA are considered as integral components of centromeric chromatin and are tightly linked to CENP-A deposition [6–9]. Their upregulation has been observed in several cancer cell lines [46], including breast cancer cells lacking BRCA1 [27]. Since we found that BRCA1 preserves centromeric chromatin identity by resolving R-loops, we hypothesized that increased cenRNA expression in BRCA1-deficient cells also correlates with increased R-loops. This was tested by using RT-qPCR to monitor cenRNA transcript levels in U-2OS cells in the presence or absence of BRCA1 and of GFP-hRNase H1 (Fig. 3E). CenRNA (mCbox) transcripts levels were normalized to control (GAPDH) transcripts levels. This revealed that BRCA1 depletion triggered a 2-fold upregulation of cenRNA which was suppressed by RNase H1 overexpression (Fig. 3E). This suggests that the increased cenRNA expression in BRCA1-deficient cells is caused by accumulation of R-loops.

Both derepression of cenRNA and CENP-A depletion have been correlated with chromosomal instability [10, 47, 48]. The chromosomal fragments which most likely lack a functional centromere can missegregate, forming one to several micronuclei (MN) [49]. We therefore tested whether BRCA1 silencing and R-loop accumulation mediate MN formation. The fraction of cells with MN was assessed in co-transfected U-2OS cells and scored following DAPI and CREST staining to distinguish centromere-positive MN (CREST+) from acentric MN (CREST-) (Fig. 3F). A 2.3-fold increase in the total number of MN was detected in BRCA1-depleted cells compared to control cells, with a high proportion of MN containing acentric chromosome fragments (Fig. 3F). Importantly, this increase in MN events was abolished by RNase H1 overexpression (Fig. 3F), showing that BRCA1 deficiency favors the formation of R-loop-dependent acentric MN, a hallmark of genome instability.

Altogether, our data uncovered BRCA1 as an important determinant of the maintenance of centromeric chromatin identity, through preventing aberrant accumulation of R-loops at centromeric repeats. Our findings further demonstrate that BRCA1-mediated suppression of R-loops at centromeres modulates cenRNA expression and acentric MN formation indicative of genomic instability.

### BRCA1 protects centromeres from R-loop-induced DSB

Numerous studies have shown that failure to resolve R-loops promotes the formation of deleterious DSBs [18, 50]. Chromosome breakage occurs more frequently at the (peri)centromeric regions than at other genomic regions [51]. These very large arrays of tandem DNA repeats may be considered as chromosomal fragile sites prone to DNA breaks and rearrangements [3]. We reasoned that, since persistent R-loops are prone to induce DSBs, more DSBs should be observed at centromeres in the absence of BRCA1, whereas overexpression of hRNase H1 or inhibition of transcription should prevent DSB formation.

**Fig. 4:**
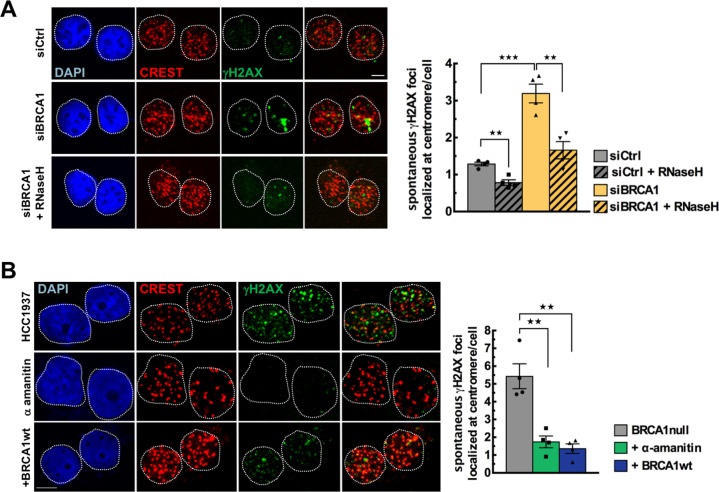
BRCA1 protects centromeres from R-loop-induced DSB accumulation. **A** Representative maximum intensity projections images showing DAPI (blue), anti-centromere Ab CREST (red) and anti-γH2AX Ab (green) signals from U-2OS cells co-transfected with siRNAs and GFP-hRNase H1 or GFP-nuc. Scale bar, 5 μm. A mask was generated to mark all centromeres on the basis of CREST staining in the image. The intensity of γH2AX signals was measured in the mask from *n* ≥ 100 cells analyzed for each condition in one biological experiment. If the γH2AX signals reached a preset threshold, they were scored as centromeric γH2AX foci. The quantitative analysis shows the mean number ± SEM of centromeric γH2AX foci/cell transfected with siRNAs and with hRNase H1 ( + RNaseH). *n* = 4 independent experiments. **p < 0.01, ***p < 0.001 (Mann–Whitney test). **B** Representative immunofluorescence images showing DAPI (blue), anti-centromere Ab CREST (red) and anti-γH2AX Ab (green) signals in HCC1937 cells treated with α-amanitin or rescued by wtBRCA1. Scale bar, 5 μm. A mask was generated to mark all centromeres on the basis of CREST staining in the image. The intensity of γH2AX signals was measured in the mask from *n* ≥ 50 cells analyzed for each condition in one biological experiment. If the γH2AX signals reached a preset threshold, they were scored as centromeric γH2AX foci. The quantitative analysis shows the mean number ± SEM of γH2AX foci localized at centromere/cell. *n* = 4 independent experiments. **p < 0.01 (Mann–Whitney test).

To test this hypothesis, we used IF to analyze the signal intensity of γH2AX, a marker of DNA breaks, and centromeres labeled by CREST serum in GFP-sorted cells co-transfected with siRNAs and plasmids expressing control GFP-nuc or GFP-hRNase H1 (Fig. 4A). In control undamaged U-2OS cells, the levels of centromeric γH2AX foci was very low (1.03 ± 0.13 centromeric γH2AX focus/cell) but represented more than half of the total number of spontaneous γH2AX foci (1.71 ± 0.2 γH2AX foci/cell) (Fig. S3C). In contrast, we detected only 6% of the total γH2AX foci at centromeres 30 min following X-ray irradiation, which induces randomly distributed DNA damage throughout the genome. This reveals the large contribution of the intrinsic fragility of the centromeric regions to spontaneous DNA damage (Fig. S3C). Depletion of BRCA1 increased spontaneous centromeric γH2AX foci by 2.5-fold (Fig. 4A). Strikingly, GFP-hRNase H1 overexpression abolished the induction of centromeric γH2AX foci in BRCA1-depleted cells (from 2.6 to 1.4 γH2AX foci; Fig. 4A), indicating that BRCA1 loss favors the formation of R-loop-dependent DSBs at α-SAT repeats.

To further confirm that the suppression of R-loops at centromeres by BRCA1 is essential to genome stability, we examined centromeric γH2AX foci in HCC1937 cells and their wtBRCA1-rescued counterparts (Fig. 4B). BRCA1-null cells showed significantly more centromeric γH2AX foci than wtBRCA1 cells (5.32 ± 0.88 centromeric γH2AX foci in HCC1937 cells versus 1.33 ± 0.33 in BRCA1-rescued HCC1937 cells) (Fig. 4B). Again, spontaneous centromeric γH2AX foci represented more than half of the total foci counted per cell (Fig. S3D). Remarkably, α-amanitin treatment decreased the percentage of spontaneous centromeric γH2AX foci in BRCA1-null cells by 3.7-fold, supporting the notion that spontaneous centromeric breakage observed in the absence of BRCA1 is derived from active transcription-mediated formation of R-loops (Fig. 4B). To strengthen our data, we measured the relative γH2AX abundance by ChIP at different α-SAT repeats in HCC1937 cells versus BRCA1-rescued HCC1937 cells. Previous data suggest that γH2AX signals associated with R-loops and detected by ChIP are mainly a reflection of the presence of single-stranded DNA breaks as a source of DNA damage [22]. Analysis of the γH2AX ChIP data after normalization against H2AX data revealed that the high amount of DNA breaks detected at centromeres results from BRCA1 deficiency (Fig. S3E).

Altogether, our findings demonstrate that the accumulation of centromeric R-loops favored by BRCA1 deficiency induces spontaneous DNA breaks at α-SAT repeats.

### BRCA1 prevents Rad52-dependent recombination between satellite repeats

**Fig. 5:**
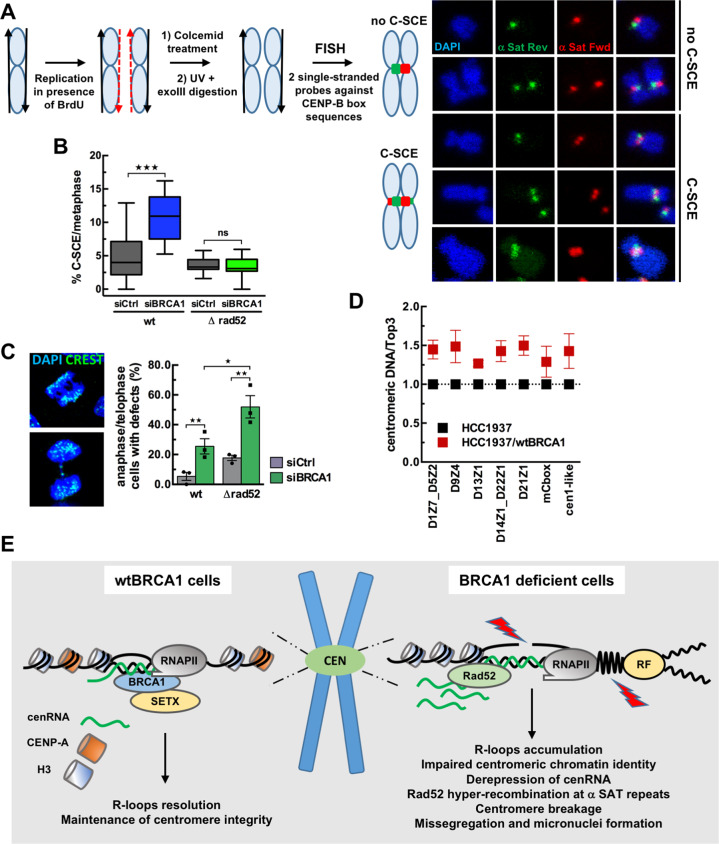
BRCA1 prevents Rad52-dependent recombination between satellite repeats. **A** Scheme describing the α−SAT repeats recombination monitored by CO-FISH in siRNA-treated U-2OS and ΔRad52 U-2OS cells. Cells are allowed to replicate once in the presence of BrdU, giving rise to chromosomes with one BrdU-containing chromatid (red dashed line). After colcemid treatment to arrest cells in prometaphase, fixation of cells on slides and Hoechst staining, the BrdU-containing DNA strands are degraded following UV exposure and digestion with ExoIII. Metaphases are then hybridized with strand-specific PNA probes against CENP-B box sequences. In the absence of sister-chromatid exchange (SCE) at α-SAT repeats, only one chromatid per chromosome will show a signal upon hybridization with either the leading or the lagging probes (no C-SCE). If SCE has occurred within α-SAT repeats, labeling will split between the sister-chromatids, giving rise to two signals per chromatid (C-SCE). Examples of CO-FISH signals showing no centromeric sister-chromatid exchange between repeats (no C-SCE) and CO-FISH patterns showing recombination between repeats (C-SCE) after hybridization with single-stranded centromeric PNA probes. **B** The box plot shows the distribution of the percentage of C-SCE per metaphase from *n* ≥ 18 metaphases analyzed for each condition in one biological experiment. ***p < 0.001; ns, not significant (Mann–Whitney test). Similar results were obtained from two additional independent experiments. **C** Representative immunofluorescence images displaying mitotic defects in siRNA-treated U-2OS cells stained with DAPI and CREST (green). The quantitative analysis shows the mean percentage ± SEM of cells with mitotic defects from *n* ≥ 12 anaphase/telophase cells analyzed for each condition in one biological experiment. *n* = 3 independent experiments. *p < 0.05, **p < 0.01 (Mann–Whitney test). **D** Analysis of α-SAT repeats copy number by qPCR in HCC1937-derived cell lines. Chart shows the relative quantity of centromeric DNA from several chromosomes in wtBRCA1-reconstituted cells as compared to BRCA1null cells as mean ± SEM. *n* = 4 independent experiments. **E** Model illustrating how BRCA1 prevents R-loop-associated centromeric instability. R-loops favor the recruitment of BRCA1 at centromeres to maintain centromeric chromatin identity, to antagonize centromere breakage, and to stabilize repetitive sequences, avoiding centromere-driven chromosome instability and missegregation.

In mammalian cells, centromeres are highly recombinogenic compared to the rest of the genome [35]. BRCA1 promotes HR-mediated repair of DSB and protects stalled replication forks from nucleolytic degradation [21]. We thus predicted that the increase in spontaneous centromeric DSB in BRCA1null cells is likely a combination of increased fork stalling due to persistent R-loops and decreased non-crossover recombination events, leading to enhanced crossover products. To monitor centromeric sister-chromatid HR in BRCA1-depleted U-2OS cells, we used a chromosome orientation fluorescence in situ hybridization assay (CO-FISH) which measures sister-chromatid exchanges (SCE) at specific endogenous loci such as centromeres (Fig. 5A) [36]. We used a set of strand-specific PNA probes differentially labeled which hybridize either with the leading strand (5′–3′) or with the lagging strand (3′–5′) of α-SAT repeats [36]. These probes are complementary to the CENP-B box sequences present within the centromeric repeats of all autosomes and X chromosome [52]. In the absence of SCE between centromeric sequences (C-SCE), each sister-chromatid shows a distinct fluorescent signal after hybridization (Fig. 5A, no C-SCE). In contrast, if crossover has occurred between α-SAT repeats, both signals of unequal intensity split between sister-chromatids (Fig. 5A, C-SCE). Consistent with our prediction, BRCA1 depletion led to a significant >2-fold increase in the percentage of C-SCE observed per metaphase (Fig. 5B), supporting the notion that BRCA1 is necessary to limit spontaneous crossovers between α-SAT sequences.

To further characterize the hyper-recombination phenotype associated with BRCA1 deficiency, we determined the frequency of recombination of α-SAT repeats in a Rad52 knock-out (ΔRad52) U-2OS cells [31] (Fig. S3F). Rad52 is one of the alternative recombination factors in BRCA1-deficient cells [53]. Depletion of BRCA1 did not affect proliferation of ΔRad52 U-2OS cells during the experiment time-course (Fig. S3G). Suppression of Rad52 expression in combination with BRCA1 depletion drastically reduced the frequency of spontaneous SCE observed at centromeres in the absence of BRCA1 (Fig. 5B). These results demonstrate that Rad52 mediates the increased rate of SCE observed at α-SAT sequences in the absence of BRCA1. To investigate the cellular consequences of this effect, we next assessed whether BRCA1 and Rad52 deficiencies might lead to mitotic aberrations. The presence of chromatin bridges and lagging chromosomes were scored in siRNA-transfected anaphase and telophase U-2OS and ΔRad52 U-2OS cells (Fig. 5C). As previously shown, BRCA1 [25, 26] or Rad52 [54] deficiencies caused an increase in the frequency of mitotic aberrations compared to control (Fig. 5C). Strikingly, combined inhibition of BRCA1 and Rad52 resulted in a marked increase in anaphase and telophase defects (Fig. 5C), suggesting that Rad52 may provide an essential alternative pathway for dealing with R-loops associated DNA damage in the absence of BRCA1.

Finally, given that increased recombination generates genetic instability and may lead to duplications or deletions, we next examined whether the high frequency of Rad52-dependent SCE found at centromeres in BRCA1-null cells was associated with centromeric DNA instability. Using a PCR-based methodology to analyze centromere genomics [55], we measured the abundance of α-SAT repeats on several human chromosomes in BRCA1-null and wtBRCA1-reconstituted HCC1937 cells. For all markers of centromeres tested, we found lower copy numbers of α-SAT in BRCA1-null cells than in wtBRCA1-complemented cells (Fig. 5D), supporting the view that the prolonged absence of BRCA1 favors the deletion of centromeric DNA sequences.

Altogether, our data reveal that BRCA1 is critical for maintaining the stability of human centromeric tandem repeats in undamaged cells and hence contributes to proper chromosome segregation.

## Discussion

Here, we unveiled a key role for BRCA1 in protecting human centromeres from the accumulation of deleterious R-loops and their associated genomic instability and in maintaining centromere identity, which is crucial for the faithful inheritance of the genetic material after cell division.

Using DRIP and immunofluorescence, we showed that human centromeric repeats are prone to R-loop formation during interphase, a behavior which was previously observed in yeast [56, 57], for other repeats throughout the human genome [32] and during mitosis [17]. In turn, we revealed that centromeric chromatin association of BRCA1 is promoted by R-loops in undamaged cells. R-loops-dependent recruitment of BRCA1 might be direct as supported by recent findings showing that purified recombinant BRCA1 recognizes DNA:RNA hybrids as assessed in vitro by electrophoretic mobility shift assay with DNA:RNA substrates [58]. Alternatively, since BRCA1 is known to be associated with RNAPII during transcriptional elongation [59], BRCA1 could also be retained at centromeric R-loop-dependent RNAPII pausing [23].

Our results further showed that one of the important functions of BRCA1 at centromeres is to antagonize R-loop accumulation. This is very likely mediated by the helicase SETX, a known BRCA1 partner [22, 28] and a factor limiting R-loops accumulation [14], since we observed that SETX recruitment at centromeres was reduced upon BRCA1 depletion. Then, by counteracting persistent centromeric R-loops, BRCA1 prevents a critical barrier to replication fork under physiological conditions which can have severe consequences at repetitive sequences.

Next, we found that the accumulation of R-loops at α-SAT repeats reduces CENP-A abundance at centromeres of BRCA1-deficient cells. Since impairment of the centromeric replication causes defects in CENP-A deposition [60], one hypothesis for decreased CENP-A levels with increased RNA:DNA hybrids is that unscheduled R-loops may impede the progression of replication forks [19]. Interestingly, a recent study, using a system allowing rapid removal of endogenous CENP-A-containing nucleosomes, established that CENP-A represses R-loop formation during DNA replication, facilitating fork progression [48]. This suggests that R-loops accumulation induced by BRCA1 deficiency alters CENP-A chromatin which in turn contributes to stabilize R-loops at centromeres during S phase. Altogether, our data shed new light on the link between BRCA1 deficiency and chromosomal instability, via the destabilization of CENP-A, epigenetic mark of the centromere integrity, when unscheduled accumulation of deleterious R-loops occurs at these key regions for chromosome stability.

In addition, we found that the persistence of centromeric RNA:DNA hybrids favors the transcription of α-SAT repeats. This is consistent with R-loops functioning as epigenetic marks, by altering chromatin and transcription [15, 61]. Thus, the suppression of R-loops at transcribed centromeric repeats by BRCA1 may also contribute to the silencing of satellite repeats as previously shown by Zhu et al. [27]. Since the activation of centromeric repeats transcription may result in the rapid delocalization of CENP-A molecules from their default location [62, 63], this also raises a possibility that CENP-A decrease observed upon BRCA1 deficiency is facilitated by cenRNA de-repression.

Finally, we demonstrated that BRCA1 deficiency creates a context favorable for the accumulation of spontaneous R-loop-mediated DSB at centromeres. Under these conditions, DNA breaks occur in highly repetitive satellite regions, which are extremely vulnerable to faulty repair. We found that this increased level of centromeric breakage directly correlates with enhanced SCE in BRCA1-depleted cells. This reveals that BRCA1 deficiency increases crossover recombination between α-SAT tandem repeats and thereby centromeric instability. Importantly, we identified Rad52 as a key player in these spontaneous recombination events in the absence of BRCA1. Interestingly, R-loops contribute in recruiting Rad52 [31], which functions in Break-Induced Replication (BIR), a repair pathway for stalled DNA replication fork which was shown to bypass R-loops in yeast [64, 65]. BIR is a highly mutagenic pathway of HR when it relies on strand invasion between repetitive sequences and can result in gain or loss of genetic information [18]. In agreement, we found a loss of α-SAT repeats in BRCA1-null cancer cells compared to that in wtBRCA1-complemented cells, indicating that BRCA1 is critical for centromeric repeats stability. Interestingly, we found that combined inhibition of BRCA1 and Rad52 which abolishes SCE at centromere strongly increases missegregation, suggesting that Rad52 contributes to an essential salvage pathway in BRCA1-depleted cells. Moreover, increased SCE have also been observed at human centromeric repeats following CENP-A depletion, showing that the integrity of the centromeric chromatin is crucial for the stability of the underlying α-SAT sequences [36, 48] and strongly suggesting that CENP-A reduction following BRCA1 loss may precede SCE events.

R-loop accumulation can lead to genome instability [19]. A dysfunctional centromere with an excess of R-loops could missegregate, forming MN. In agreement, we observed a higher frequency of R-loop associated MN in BRCA1-depleted cells. While centromeric R-loops have been recently shown to be beneficial for faithful mitosis [17], our study indicates that their aberrant accumulation during interphase induces centromere instability, illustrating the necessary homeostasis of R-loops at centromeres throughout the cell cycle.

Altogether, our data uncover a central role played by BRCA1 and its associated proteins in the maintenance of centromere integrity and identity, by counteracting deleterious R-loop accumulation, maintaining centromeric chromatin identity, antagonizing centromere breakage, mutagenic recombination, missegregation and formation of micronuclei, thereby stabilizing repetitive sequences to avoid centromere-driven chromosome instability (Fig. 5E). Because DNA breakage at centromere can lead to gross chromosomal rearrangements, which cause cell death and genetic diseases including cancer [51, 66], our findings have important implications for understanding both the organization of the centromere and how its instability is linked to tumorigenesis.

## Supplementary information

Figure S1

Figure S2

Figure S3

Table S1

Table S2

## Data Availability

All the relevant data are available from the corresponding author on reasonable request.
