# Supplementary figures and images for: BRCA1 prevents R-loop-associated centromeric instability
Source: Cell Death Dis. 2021 Oct 1;12(10):896. doi: 10.1038/s41419-021-04189-3 (PMC8486751; doi:10.1038/s41419-021-04189-3)

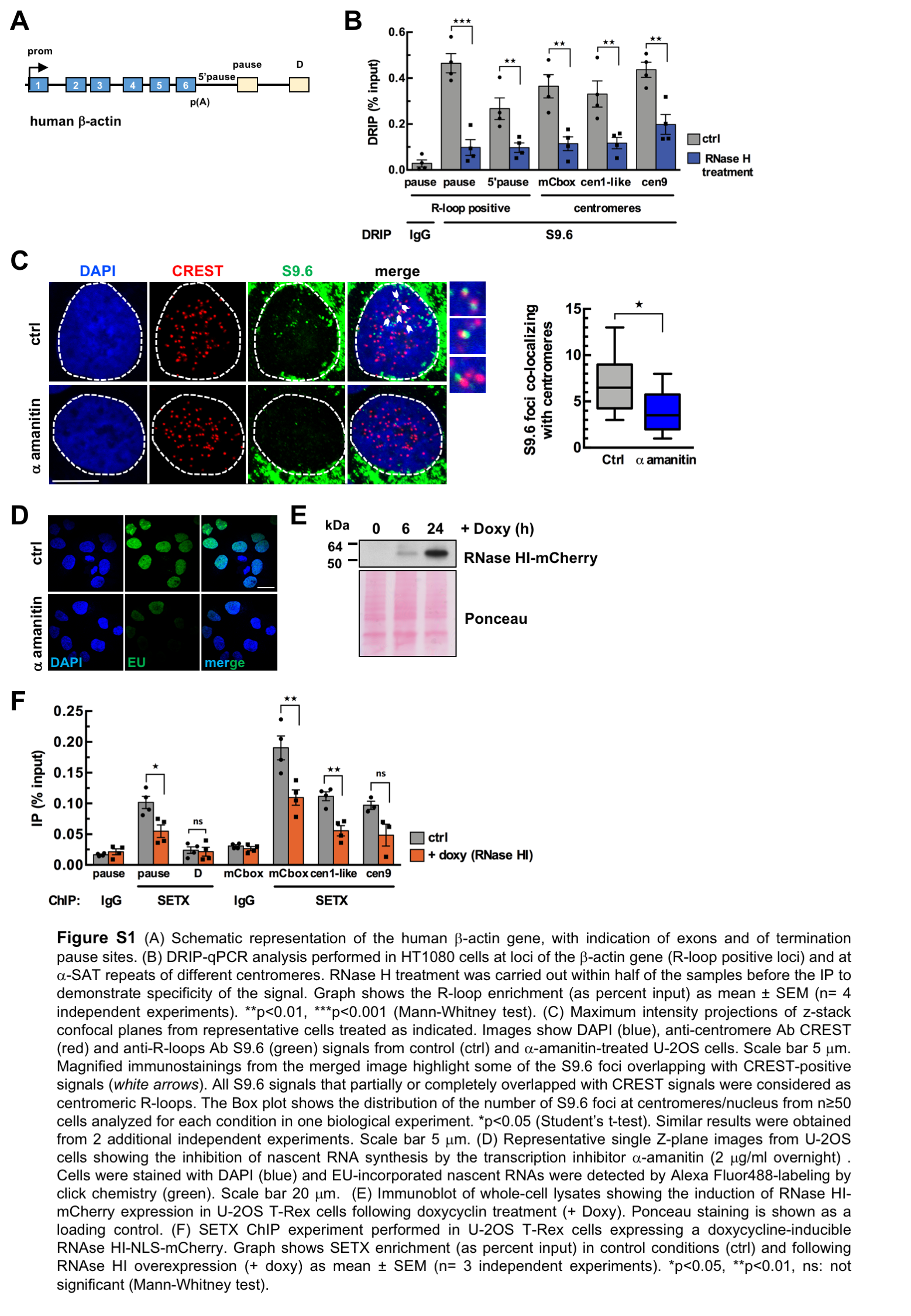

Supplement: Supplementary file 1 — Figure S1 [file 41419_2021_4189_MOESM1_ESM.tif]

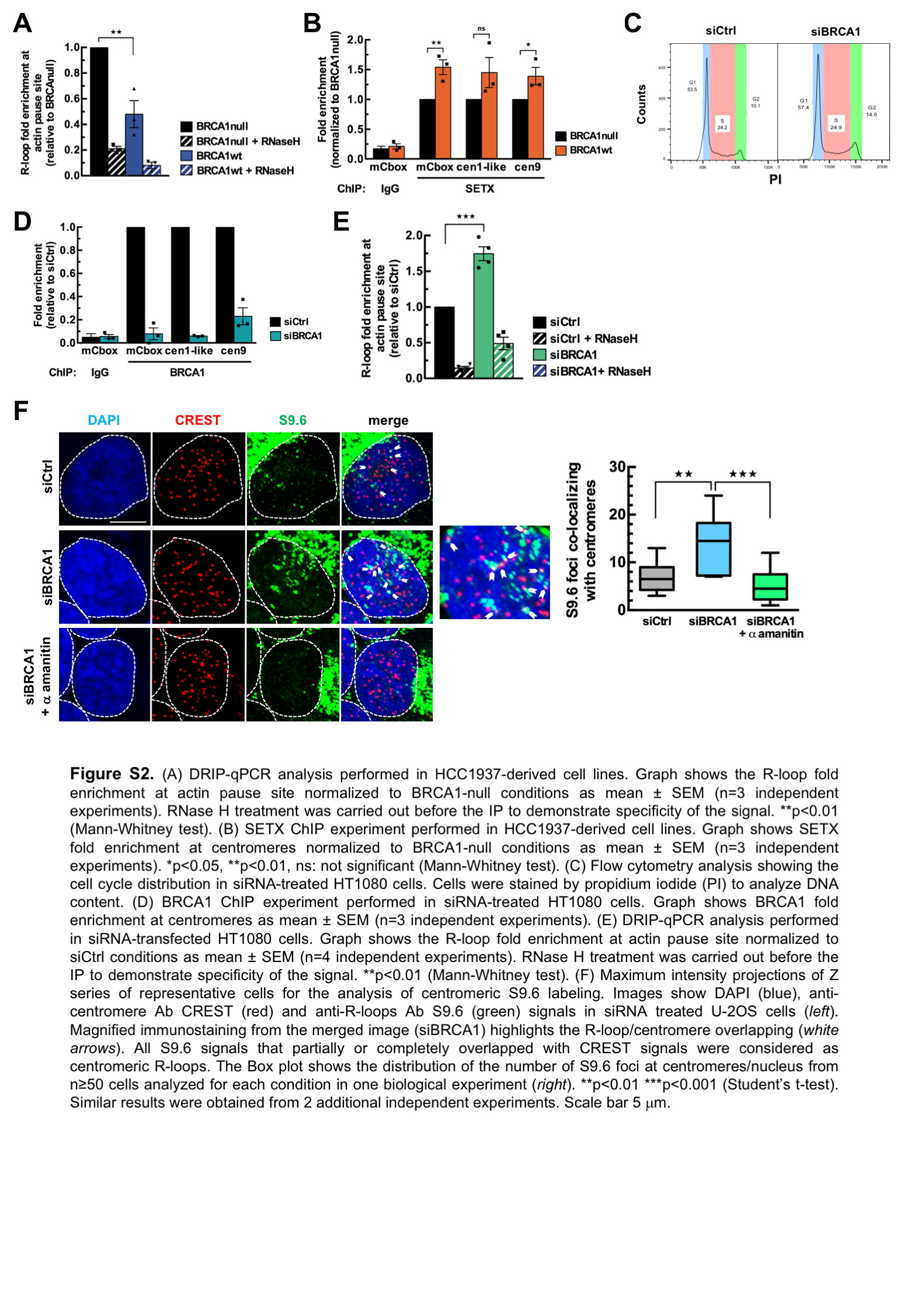

Supplement: Supplementary file 2 — Figure S2 [file 41419_2021_4189_MOESM2_ESM.tif]

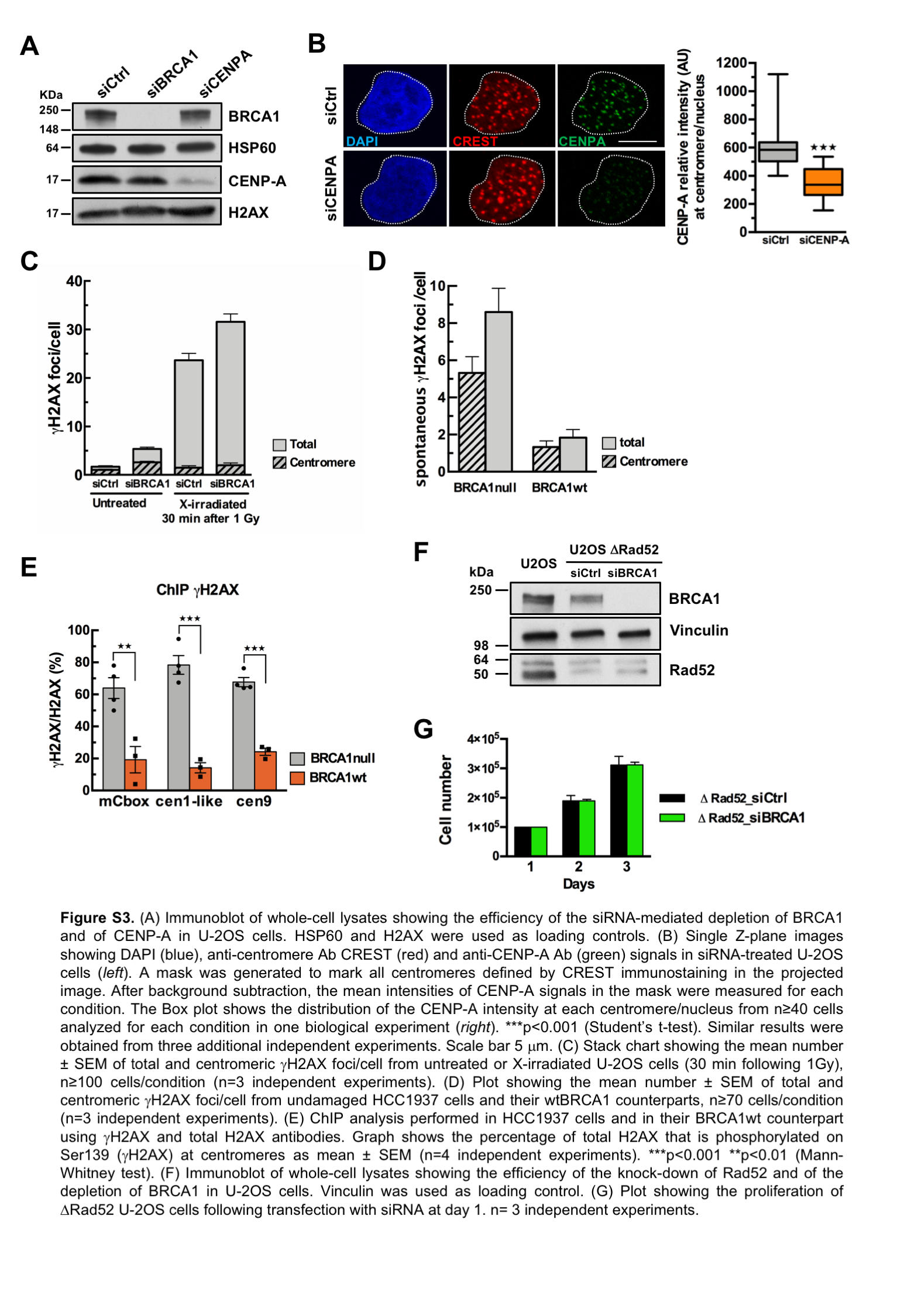

Supplement: Supplementary file 3 — Figure S3 [file 41419_2021_4189_MOESM3_ESM.tif]
