# Supplementary material for: BRCA1 prevents R-loop-associated centromeric instability
Source: Cell Death Dis. 2021 Oct 1;12(10):896. doi: 10.1038/s41419-021-04189-3 (PMC8486751; doi:10.1038/s41419-021-04189-3)
Supplement: Supplementary file 4 — Table S1 [file 41419_2021_4189_MOESM4_ESM.docx]

| **PRIMARY ANTIBODY** | **SOURCE** | **IDENTIFIER** | **DILUTION OR QUANTITY** |
| --- | --- | --- | --- |
| Mouse monoclonal anti-actin (C4) | Millipore | MAB1501 | 1/10000 for WB |
| Rabbit polyclonal anti-BRCA1 | Bethyl | A300-000A | 4 μg/ChIP |
| Mouse monoclonal anti-BRCA1 (D-9) | Santa Cruz | sc-6954 | 1/200 for WB |
| Mouse monoclonal anti-CENPA (3-19) | Abcam | ab13939 | 2 μg/ChIP  1/200 for IF |
| Rabbit polyclonal anti-CENPA | Cell Signaling Tech. | 2186S | 1/1000 for WB |
| CREST serum | Strasbourg Hospital, France | from patients | 1/1000 for IF |
| Mouse monoclonal anti-GFP | Roche | 11814 | 1/1000 for WB |
| Rabbit polyclonal anti-H2AX | Abcam | Ab11175 | 1/10000 for WB  1 μg/ChIP |
| Mouse monoclonal anti-pH2AX (Ser139) | Millipore | 05-636 | 1/1000 for IF  2 μg/ChIP |
| Mouse monoclonal anti-H3 | Active Motif | MABI 0301 | 1 μg/ChIP |
| Mouse monoclonal anti-HSP60 (LK2) | Sigma-Aldrich | H3524 | 1/2000 for WB |
| Mouse monoclonal anti-DNA-RNA hybrid S9.6 | Kerafast | ENH001 | 5 μg/DRIP |
| Mouse monoclonal anti-DNA-RNA hybrid S9.6 | Purification from hybridoma | In-house | 3-6 μg/DRIP |
| Rabbit polyclonal anti-Rad52 | ABclonal | A3077 | 1/500 for WB |
| Rabbit polyclonal anti-RFP | Abcam | Ab62341 | 1/2000 for WB |
| Rabbit polyclonal anti-SETX | Novus Biologicals | NBP1-94712 | 5 μg/ChIP  1/500 for WB |
| Mouse monoclonal anti-vinculin | Santa Cruz | sc-73614 | 1/1000 for WB |
| **SECONDARY ANTIBODY** | **SOURCE** | **IDENTIFIER** | **DILUTION OR QUANTITY** |
| Alexa fluor 488 goat anti-mouse IgG | Invitrogen | A-11001 | 1/1000 for IF |
| FluoProbe 547H goat anti-human IgG | Interchim | FP-SB3110 | 1/1000 for IF |
| Alexa fluor 647 goat anti-mouse IgG | Invitrogen | A-21235 | 1/1000 for IF |
| Alexa fluor 647 goat anti-human IgG | Invitrogen | A-21445 | 1/1000 for IF |
| Peroxidase AffiniPure Goat Anti-Rabbit IgG | Jackson ImmunoResearch | **111-035-003** | 1/10000 for WB |
| Peroxidase AffiniPure Goat Anti-Mouse IgG | Jackson ImmunoResearch | **115-035-003** | 1/5000 for WB |
| Negative Ctrl IgG from rabbit | Diagenode | C15410206 | 2-5 μg/ChIP |
| Negative Ctrl IgG from mouse | Diagenode | C15400001-100 | 3-6 μg/DRIP  2-5 μg/ChIP |

**Table S1:** List of antibodies used in western blotting (WB), immunofluorescence imaging (IF), DRIP and ChIP experiments.
