# Supplementary material for: BRCA1 prevents R-loop-associated centromeric instability
Source: Cell Death Dis. 2021 Oct 1;12(10):896. doi: 10.1038/s41419-021-04189-3 (PMC8486751; doi:10.1038/s41419-021-04189-3)
Supplement: Supplementary file 5 — Table S2 [file 41419_2021_4189_MOESM5_ESM.docx]

| **NAME** | **CHROMOSOME DETECTED** | **FORWARD PRIMER** | **REVERSE PRIMER** | **PCR FRAGMENT SIZE** |
| --- | --- | --- | --- | --- |
| β actin pause | 7 | GGGACTATTTGGGGGTGTCT | TCCCATAGGTGAAGGCAAAG | 167 |
| β actin 5’pause | 7 | TTACCCAGAGTGCAGGTGTG | CCCCAATAAGCAGGAACAGA | 105 |
| β actin D | 7 | CAGTGGTGTGGTGTGATCTTG | GGCAAAACCCTGTATCTGTGA | 151 |
| Cen1-like | 1,5,19 | TCATTCCCACAAACTGCGTTG | TCCAACGAAGGCCACAAGA | 136 |
| mCbox | 21 | AGGGAATGTCTTCCCATAAAAACT | GTCTACCTTTTATTTGAATTCCCG | 195 |
| cen9 | 4,9 | CCTATGATGAAAAAGGTAATATCTTC | CTGAAAGCGCTTAAAACGTCC | 337 |
| Top3 | 17 | ACTAGGTCAGAGACCCTTACTG | CAAGGAGAGGCAGTGACAAA | 127 |
| D1Z7_D5Z2 | 1,5 | GTTCCCTTAGACAGAGCAGATTT | CAACGCAGTTTGTGGGAATG | 140 |
| D9Z4 | 9 | GGAGAAGCATTCTCAGGAACTT | GTCCGCTTGCAGATACTACAG | 114 |
| D13Z1 | 13 | TGATGTGTGTACCCAGCT | GCTATCCAAATATCCACT | 98 |
| D14Z1_D22Z1 | 14,22 | CAATCTCAGAATCTTCTTTGGGATA | CCAAGCTATCCAAATATCCACTT | 122 |
| D21Z1 | 13,15, 21 | TGATGTGTGTACCCAGCC | GCTATCCAAATATCCACC | 98 |

**Table S2:** List of primer sequences used for qPCR experiments.
